# LLT1-CD161 Interaction in Cancer: Promises and Challenges

**DOI:** 10.3389/fimmu.2022.847576

**Published:** 2022-02-04

**Authors:** Veronique M. Braud, Aïda Meghraoui-Kheddar, Roxane Elaldi, Luciana Petti, Claire Germain, Fabienne Anjuère

**Affiliations:** ^1^Université Côte d’Azur, CNRS UMR7275, Institut de Pharmacologie Moléculaire et Cellulaire, Valbonne, France; ^2^Biomunex Pharmaceuticals, Paris, France

**Keywords:** CD161, LLT1, cancer, immune checkpoint, tertiary lymphoid structure (TLS)

## Abstract

The success of immune checkpoint therapy in cancer has changed our way of thinking, promoting the design of future cancer treatments that places the immune system at the center stage. The knowledge gained on immune regulation and tolerance helped the identification of promising new clinical immune targets. Among them, the lectin-like transcript 1 (LLT1) is the ligand of CD161 (NKR-P1A) receptor expressed on natural killer cells and T cells. LLT1/CD161 interaction modulates immune responses but the exact nature of the signals delivered is still partially resolved. Investigation on the role of LLT1/CD161 interaction has been hampered by the lack of functional homologues in animal models. Also, some studies have been misled by the use of non-specific reagents. Recent studies and meta-analyses of single cell data are bringing new insights into the function of LLT1 and CD161 in human pathology and notably in cancer. The advances made on the characterization of the tumor microenvironment prompt us to integrate LLT1/CD161 interaction into the equation. This review recapitulates the key findings on the expression profile of LLT1 and CD161, their regulation, the role of their interaction in cancer development, and the relevance of targeting LLT1/CD161 interaction.

## Introduction

Natural killer (NK)-cell receptors (NKRs) are found expressed on the surface of NK cells and T cells where they contribute to regulate the threshold of activating and inhibitory signals, thus actively participating in the regulation of immune responses. Within these NKRs, a cluster encoded at the NK gene complex on chromosome 12p13 in humans and chromosome 6 in mice, belong to the C-type lectin-like superfamily (1, 2). They bear a C-type lectin-like domain which has lost the ability to bind Ca^2+^ and is involved in protein-protein interaction rather than carbohydrates binding. They comprise the activating NKG2D receptor expressed as homodimer and binding the stress-induced molecules MICA/B and ULBP (3, 4), the heterodimers CD94/NKG2 receptors binding HLA-E, which deliver inhibitory signals when associated with NKG2A and activating signals when associated with NKG2C (5–7) and receptors of the NKR-P1 family whose ligands belonging to the CLEC2 clade are also located in the NKC complex (8, 9). While the genomic organization is well conserved, the number of genes varies with an extension in mouse and rat compared to human (10). *KLRB1* and *CLEC2D* are among genes that are the most duplicated. In humans, a single *KLRB1* gene encodes for NKR-P1A (CD161) (11) and a single *CLEC2D* gene encodes for LLT1 (OCIL, Clr) (12) while 6 or 4 *Klrb1* genes and 8 or 10 *Clec2* genes are present respectively in the mouse and rat NKC. These differences render difficult the studies of LLT1/CD161 interaction in mice and rat models as no functional homologue is clearly defined. In 2005, LLT1 was identified as the ligand of CD161 (13, 14). While it is admitted that LLT1/CD161 interaction plays a role in regulating immune responses in infectious diseases, autoimmunity, inflammatory conditions and cancer, the nature of these regulations remains to be fully examined. In the present review, we will recapitulate the current knowledge on LLT1 and CD161 expression profile, and their role in tissue homeostasis and pathology. We will focus on their role in cancer immune surveillance and discuss the potential of therapeutic strategies targeting this interaction.

## LLT1 Expression is Induced on Hematopoietic Cells Upon Activation

*CLEC2D* gene expression is reported primarily in hematopoietic cells. Exon skipping generates alternatively spliced transcript variants, with *CLEC2D* variant 1 coding for LLT1 (isoform 1) (15). LLT1 is the sole protein isoform expressed at the cell surface, which binds to CD161. Two transcripts variants encode for transmembrane protein isoforms 2 and 4 which remain intracellular and their functions are still to be elucidated. So far, there is no solid evidence for a soluble form of LLT1. A transcript variant encodes for a putative soluble LLT1 but 20 N-terminal amino acids prevent efficient translation and production of the protein (15). In addition, the generation of soluble LLT1 by proteolysis has not been demonstrated. Only one study reported the presence of soluble LLT1 in the sera of rheumatoid arthritis and spondyloarthropathy patients, without correlation with general inflammation and disease activity (16). However, the specificity of the detection kit based on polyclonal antibodies used in this study needs to be fully assessed before drawing definitive conclusions. Indeed, CLEC2D being highly homologous to the other members of the CLEC2 clade (CLEC2A/KACL, CLEC2B/AICL and CLEC2C/CD69), misinterpretation of some of the data can be found in the literature. First, the proliferation-induced lymphocyte-associated receptor (PILAR) aligns with CLEC2A sequence and does not bind to CD161 as claimed in (17) but to NKp65 expressed by NK cells upon allogeneic and IL-2 stimulation (18, 19). In agreement, CLEC2A/KACL-Fc multimers failed to bind to CD161 and CLEC2A/KACL was not expressed by activated T cells (15, 18). Second, the use of the non-specific antibody clone 4C7 is misleading. It was demonstrated that 4C7 binds to all the CLEC2D isoforms, to CLEC2A/KACL and not to CLEC2B/AICL and CLEC2C/CD69 (15, 20). It also recognizes a non-identified cell surface molecule expressed in vaccinia virus western reserve-infected B cell lines (21). Thus, the 4C7 mAb clone lacks specificity and should be renamed as an anti-CLEC2D/CLEC2A antibody. Third, the antibody L9.7 originally described as a specific anti-LLT1 mAb (22) identified a band at a different molecular weight than the expected one (15, 20) and failed to bind to LLT1-expressing BA/F3 transfectants (20). These findings thus question the reported role of LLT1 as an activating receptor triggering NK cell secretion of IFN-γ upon cross-linking with L9.7 antibody (22). Altogether, such observations highlight the need to test all the anti-LLT1 antibodies generated for cross-reactivity with the other members of the CLEC2 clade before use.

**Figure 1:**
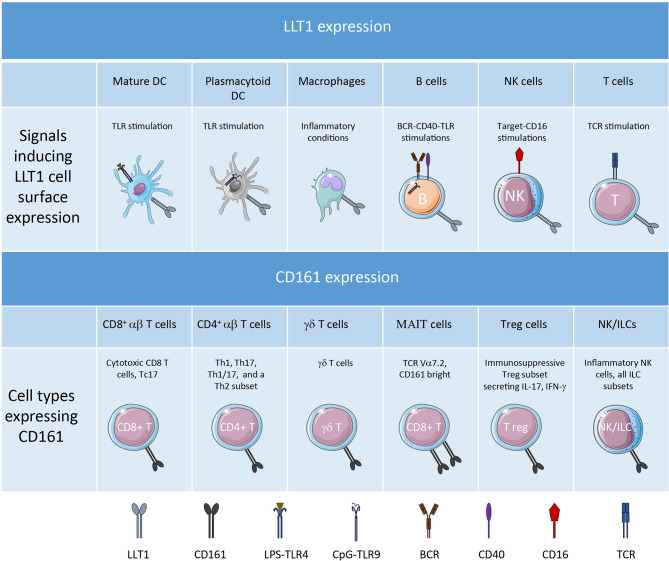
LLT1 and CD161 expression profile.

LLT1 is expressed as a disulfide-bonded homodimer and binds with low affinity (K_D_=48μM) to homodimers of CD161 (23–25). This is the weakest binding affinity compared to CLEC2B/AICL binding to NKp80 with a K_D_ = 2.3 μM and CLEC2A/KACL binding to NKp65 with a K_D_ = 11 nM (18, 26). LLT1 expression was reported on lymphoid and myeloid cells (**Figure 1**). LLT1 is not expressed in resting hematopoietic cells but is induced upon activation and is associated with proliferation. Several activating signals can induce its expression, including TCR and BCR cross-linking, TLR activation, costimulatory signals, and cytokine stimulation (20, 27, 28). LLT1 is not expressed on immature dendritic cells (DC) and is induced on TLR-activated DC and plasmacytoid DC (pDC). Strong induction was reported upon LPS stimulation of monocyte-derived DC and CpG stimulation of pDC. On B cells, LLT1 is induced upon cross-linking of the BCR together with CD40 and upon TLR stimulation. Interestingly, LLT1 expression is further increased by addition of IFN-γ which synergizes with TLR and/or BCR activation on B cells and DC. Stimulation of T cells by PHA and anti-CD3 also triggers LLT1 which is detected solely on proliferating cells. On NK cells, LLT1 can be induced upon target stimulation and cross-linking of CD16. Importantly, the induction of LLT1 requires strong and prolonged activation as opposed to CD69 that is rapidly induced upon activation of lymphoid cells. Under physiological conditions, LLT1 is primarily found expressed on centroblast and centrocyte B cells in the germinal centers (GC) of lymph nodes or tonsils, and LLT1 expression is predominant in centroblasts, consistent with their higher proliferation rate (29, 30). In fetal tissues, LLT1 is expressed by intestinal tissue-resident macrophages (31). LLT1 has also been reported to be expressed by cells of the monocyte-macrophage lineage in bones and is involved in inhibition of osteoclast formation (32). And LLT1 is expressed on chondrocytes of the articular cartilage and participates in their protection from NK cell cytotoxicity (33). In pathological contexts, LLT1 has been reported to be expressed on EBV and HIV-infected B cells (27), Dengue virus-infected CD14^+^CD16^-^ myeloid cells (34), hepatitis B virus-infected liver in correlation with viral replication (35), RSV-infected lung epithelial cells (36), monocytes of synovial fluid and macrophages within synovial tissue of patients with rheumatoid arthritis (16). LLT1 is also expressed in cancer and its expression will be fully described later in this review. The molecular mechanisms behind the regulation of LLT1 expression is still poorly understood and it is also important to bear in mind that the presence of transcripts does not guarantee protein expression as seen in resting PBMCs where low level of *CLEC2D* transcripts were measured but no LLT1 protein was detected (27).

## CD161 is Expressed on a Variety of NK/ILC and T Cell Subsets

Like LLT1, CD161 (NKR-P1A) is a C-type lectin-related type II transmembrane protein forming disulfide-linked homodimers (11). Its expression has been detected on the vast majority of NK cells which represent the main subset of group 1 innate lymphoid cells (ILC1), on most of the other subsets of ILCs and on a significant proportion of various conventional and unconventional T cell subsets (**Figure 1**). With the extensive characterization of the heterogeneity of NK and T cell subsets provided by recent developments in flow cytometry and single cell RNA-sequencing, we can now better apprehend the diversity of CD161^+^ NK and T cells.

CD161 is expressed early on in NK cell ontogeny. The early phases of NK cell development occur in the bone marrow and in the secondary lymphoid tissues. CD161 is expressed on stage 2 pre-NK cells and on stage 3 immature NK cells in secondary lymphoid organs (SLOs) and is maintained on stage 4-6 mature NK cells (37). Its expression decreases in adaptive NK cells generated in response to environmental stimuli (38) and is upregulated by IL-12 (39). CD161 acts as an inhibitory receptor on mature NK cells, inhibiting NK cell cytotoxicity and cytokine secretion (13, 14). It may have a dichotomous function in immature NK cells where it was found to control CXCL8 release (40). Besides NK cells, CD161 has also been detected on most ILCs, notably on ILC2 and ILC3 but its regulatory function still needs to be investigated (41).

Among T cells, CD161 was initially reported on memory/effector CD4^+^ and CD8^+^ αβ T cells, γδ T cells and NKT cells (11, 42–45). While most T cells express an intermediate level of CD161, a subset of CD8^+^ T cells express high level of CD161 and relate to MR1-restricted mucosal-associated invariant T (MAIT) cells which recognize microbial vitamin B metabolites (46–48). In adults, the majority of circulating CD161^bright^ CD8^+^ T cells are MAIT cells bearing the invariant TCR α chain (Vα7.2) and a semi-invariant TCR β usage while a minority bears polyclonal TCR Vα7.2^-^ chains (49). The diversity of conventional and unconventional T cells expressing CD161 infers diversity of functions, including granzyme and perforin-mediated cytotoxicity (50) and cytokine production, primarily IL-17, IFN-γ and TNF-α. Indeed, CD161 has been associated with Th17 and Tc17 phenotypes (51–53), with Th1 and Th1/17 cells (42, 54, 55) and also with a subpopulation of Th2 cells (56). In addition, CD161 was found expressed by minor populations of FoxP3^+^ regulatory T cells with immunosuppressive functions and producing IL-17 and IFN-γ cytokines (57). Interestingly, CD161 has been reported to be expressed on T cells with enhanced effector functions. The common features of these T cells expressing CD161 are their memory/effector phenotype and their rapid response upon antigen encounter, which underlines their central role in bridging innate and adaptive immune responses. These CD161^+^ T cells are abundant in tissues. They comprise not only MAIT cells associated with mucosal tissues but also αβ and γδ T cells making up to half of the T cells in the intestine (58) or liver (53, 59). CD161 may play an active role in this preferential homing as it was shown to enhance transendothelial migration of CD4^+^ T cells *in vitro* (60). Depending on the context, these CD161^+^ T cells could be pathogenic such as in allergic patients (56) or in multiple sclerosis (61) or of good prognosis in several human cancers (54, 62, 63).

While CD161 engagement on NK cells triggers inhibition, the signal delivered by CD161 in T cells is currently unclear and controversial. Several studies have shown costimulation of conventional T cells, NKT cells and MAIT cells upon CD161 engagement simultaneously with anti-CD3 and/or anti-CD28 antibodies (mAbs) coated either on plates, beads or FcR-expressing targets (13, 27, 45, 49, 63). Costimulation triggered an increased proliferation of T cells and increased production of IFN-γ and TNF-α. Other studies described the delivery of a co-inhibitory signal upon co-engagement of the TCR by either plate-bound anti-CD3 and/or anti-CD28 mAbs or a genetic inactivation of *KLRB1* in T cells. Coengagement of CD161 was found to inhibit TNF-α production by CD8^+^ T cells (20), IFN-γ and TNF-α production by MAIT cells (64) and IFN-γ production by fetal small intestine CD4^+^ T cells (31). CD161 blockade or inactivation was found to enhance T cell killing of gliomas and to favor the control of tumor growth *in vivo* (65). Lastly, other studies found no significant effect using conventional T cells (20, 31), or antigen specific T cells stimulated *in vitro* with mAb cross-linking and inactivation of *KLRB1* in T cells (62). The diverse nature of T cells and experimental settings analyzed may explain discrepancies and further studies are needed to fully apprehend the function of CD161 in T cells. Signal transduction *via* CD161 does not involve the adaptor molecules usually associated with NKRs like DAP12, DAP10, or CD3ζ. The cytoplasmic tail of CD161 was found to interact with the acid sphingomyelinase (aSMase), a lipid hydrolase that degrades sphingomyelin into ceramide (63). Ligation of CD161 triggers aSMase activation, the catalysis of sphingomyelin into ceramide and the activation of PI3K-PKB/Akt-1 pathway (40, 63). aSMase controls cellular levels of sphingomyelin and ceramide which regulates downstream signaling pathways involved in T cell activation, differentiation and apoptosis, thus playing a key role in immune homeostasis (66). aSMase interaction with CD161 may thus trigger a variety of responses linked to different cellular levels of ceramide (63).

## LLT1/CD161 Interaction Regulates Immune Cell Responsiveness

The physiological role of LLT1/CD161 interaction may be considered at multiple levels.

Along with other NKRs, LLT1/CD161 interaction seems to fine-tune the responsiveness of NK cells, downmodulating effector responses and being involved in NK cell peripheral self-tolerance, independently of MHC class I. If LLT1 is viewed as a self-ligand not expressed under resting conditions and upregulated upon activation, the level of expression of CD161 also appears to be modulated by infection and inflammation. Indeed, high levels of CD161 have been associated with pro-inflammatory NK cells showing high cytokine responsiveness (67), while reduced levels regulated by epigenetic changes in DNA methylation (38) are detected on NK cells with a memory phenotype (68). Signaling pathways still needs to be unraveled and several points must be taken into account. First, it was shown that LLT1 can also be expressed on NK cells, upon activation by targets or cross-linking of CD16 (27). Interaction of LLT1 with CD161 in *cis* has not been investigated but may contribute to the overall signal and may modulate the threshold of NK cell activation. Second, similarly to NKG2D downregulation upon binding of its ligands, engagement of CD161 with LLT1 triggers downregulation of CD161 (13). Impaired expression of NKG2D reduces NK cell effector functions, and it is thought to prevent NK cell hyper-responsiveness (69, 70). The outcome of CD161 downregulation is not known but one can postulate that it results in abrogating signals, similarly to NKG2D. These questions also apply to T cells in which it is not clear yet whether an inhibitory or activating signal is delivered. The observed induction of LLT1 on activated T cells concomitantly with the downregulation of CD161 upon binding to LLT1 may explain partly the conflicting results obtained on CD161 signaling in T cells. In addition, the multiple types of assays and diversity of T cells used most likely contribute to the different signals detected (13, 20, 27, 31, 45, 49, 62–65). This highlights the need to clearly identify whether CD161 is an inhibitory or an activating receptor. Alternatively, CD161 may deliver both signals depending on the context as discussed earlier in relation with cellular levels of ceramide. This hypothesis would be consistent with observations made for NKG2D which seems to mediate both inhibitory and activating signal, depending on the intensity and duration of ligand engagement (71).

**Figure 2:**
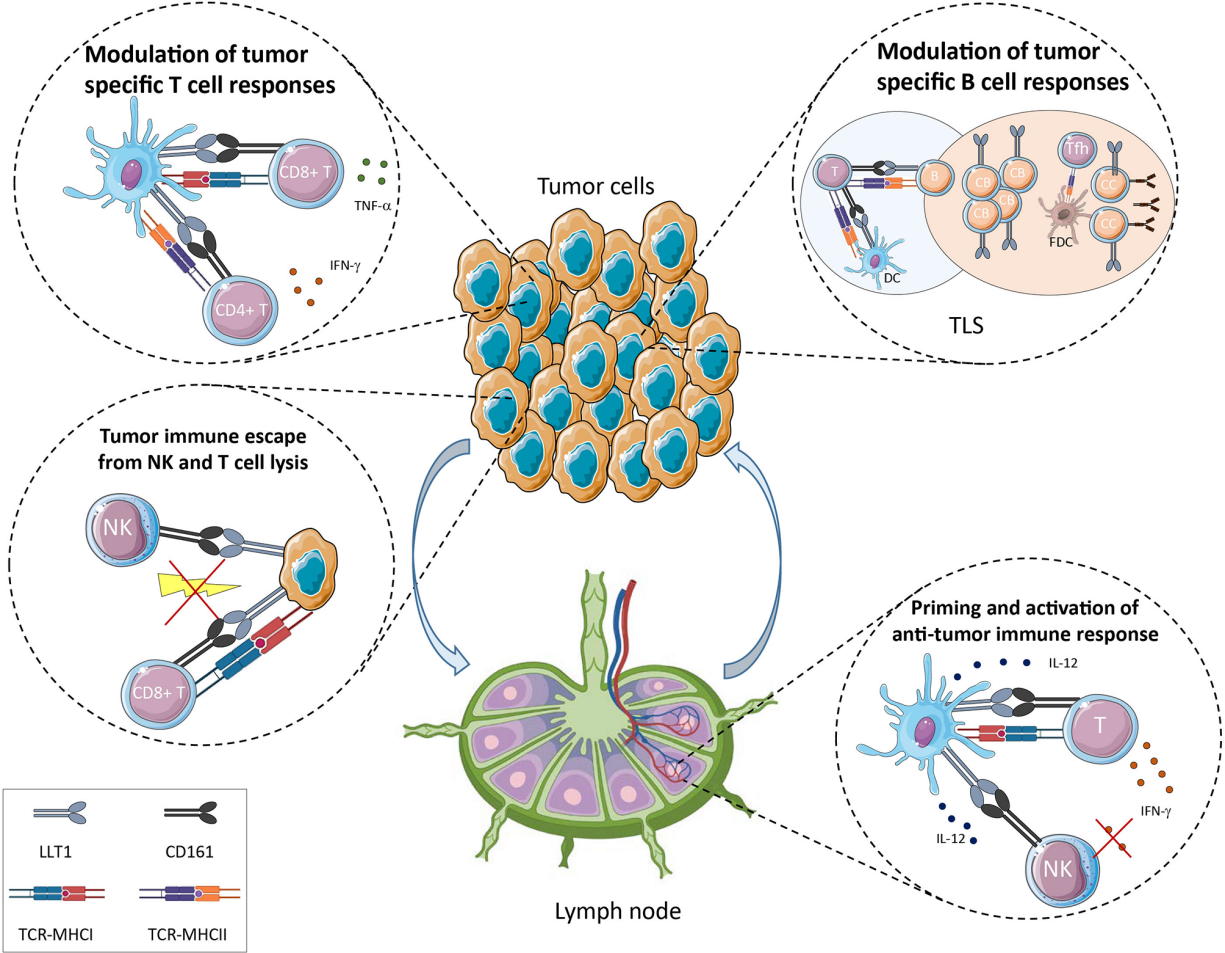
Potential role of LLT1/CD161 interaction in cancer immune surveillance.

In healthy individuals, LLT1 expression was primarily detected on GC B cells in lymph nodes and tonsils, both on centroblasts and centrocytes (29, 30) (**Figure 2**). Within SLOs, GC are structures dedicated to antibody affinity maturation, allowing the selection and expansion of B cells producing high-affinity antibodies (72–75). The initiation of this process starts with T-B interactions, at the border of T and B cell zones within SLOs. The presentation of the antigen by DC to naïve T cells in the T cell zone drives the differentiation of T cells of different lineages including pre-follicular T helper (Tfh) cells which migrate to the B cell zone, further differentiating into Tfh (76). Tfh cells interact with antigen-specific GC B cells. Follicular DC (FDC) produce chemokines that contribute to place Tfh and GC B cells into contact in the B cell zone and present antigen to sustain selection of B cells (74). LLT1/CD161 interaction is likely to play a role in these processes but because of the difficult access to human materials, it has not been possible to investigate it further. One study showed that LLT1 promoted dark zone GC B cell activation and induced the downregulation of CXCR4, suggesting that LLT1 is involved in the transition of GC B cells from the dark zone (centroblasts) to the light zone (centrocytes) (30). Because LLT1 is specifically expressed by GC B cells, it may interact with Tfh and/or FDC in the B cell zone. CD161 expression was reported on FDC by IHC and IF (30) but this result is controversial. The strong positivity for CD161 within GC observed in this study does not correlate with other published IHC and IF stainings, as well as flow cytometry phenotyping which detected CD161 on T cells outside GC and not within GC (29, 54). Interestingly, Tfh identified by a bright expression of PD-1 and CXCR5 were found to express little to no expression of CD161 (29, 30, 54). Because LLT1 interaction with CD161 triggers downregulation of CD161, it is possible that the low expression of CD161 on Tfh results from the interaction with LLT1 on GC B cells. In support of this hypothesis, a recent study that phenotyped peripheral blood PBMCs by mass cytometry detected CD161 expression in the NK cluster and in the peripheral Tfh cluster (77). These pTfh may have maintained CD161 expression because of a lack of contact with LLT1-expressing cells. Further work is needed to fully understand the role of LLT1 and CD161 in SLOs. One hypothesis is also that LLT1/CD161 interaction bridges innate and adaptive immunity. We previously identified a cross-talk between DC, NK and CD4^+^ T cells in lymph nodes and a role of CD4^+^ T cells secreting IL-2 in the activation of NK cells (78). We propose that LLT1/CD161 interaction also contributes to these processes, LLT1 being induced on mature DC (mDC) upon activation and CD161 being upregulated on NK cells and T cells by IL-12 secreted by mDC. The interaction of LLT1 with CD161 may on one hand inhibits NK cell effector functions and on the other hand costimulates T cells. This would suggest that LLT1/CD161 interaction participates in the sequential involvement of NK cells and later T cells in the initiation of adaptive immune responses, with LLT1/CD161 interaction shutting down NK cell activation while costimulating T cells (**Figure 2**).

## LLT1/CD161 Interaction and its Role in Cancer Immune Surveillance

Given the expression of LLT1 in inflammatory conditions and the wide expression of CD161 on NK/ILC and T cell populations, it is expected that LLT1/CD161 interaction is playing a role in cancer immune surveillance (**Figure 2**). This is certainly true in most human cancers where *KLRB1* coding for CD161 was found the most favorable prognostic gene in a meta-analysis of expression signatures from 18 000 human tumors across 39 malignancies (79).

Cancer development results from the accumulation of genetic mutations that deregulate cell division and provide growth advantage to tumor cells. These events are counterbalanced by innate and adaptive antitumor immune responses that eliminate emerging cancer cells. Immune surveillance and cancer immunoediting evolve towards equilibrium and tumor escape (80–82). The tumor microenvironment (TME) is a complex network regulating tumor growth that comprises tumor cells, immune cells, stromal cells, extracellular matrix, nerves, blood and lymphatic vessels. In this environment, the LLT1/CD161 interaction can play a role at all the stages of tumor development and in the modulation of associated-immune mechanisms, whether LLT1 is expressed by tumor cells or by infiltrating immune cells and whether CD161 expressing effector cells are NK/ILC or T cell populations.

### LLT1 Expression on Tumor Cells Modulates Immunity in Cancer

Because of the lack of correlation between *CLEC2D* transcripts and LLT1 protein levels, one cannot only rely on RNAseq studies to conclude on the expression of LLT1 and we need to combine transcript quantification with protein detection. Expression of LLT1 has been investigated in several human cancers and was found expressed by tumor cells and by immune cells in the TME. Mechanisms triggering LLT1 expression on tumor cells have not been thoroughly investigated but LLT1 was dectected on some tumors of hematopoietic origin.

Expression of LLT1 on tumor cells is reported for non-Hodgkin’s lymphomas (NHLs) using immunohistochemistry (IHC), immunofluorescence (IF) and flow cytometry staining (29). LLT1 is highly expressed by GC B cells and is maintained in the group of NHLs that derive from GC B cells. These include Burkitt lymphomas (BL), follicular lymphomas (FL) and GC-derived diffuse large B-cell lymphomas (GC-DLBCL). In addition, LLT1 was reported on nodular lymphocyte-predominant Hodgkin lymphomas by IHC (30). B-cell NHLs are a heterogeneous group of malignancies that are still quite difficult to differentiate and diagnose properly. LLT1 thus represents an additional biomarker that can be used to help the diagnosis of BL, FL and GC-DLBCL. The function of LLT1 on these B-cell lymphomas was investigated *in vitro* using LLT1-expressing cell lines. It was shown that its interaction with CD161 on the cell surface of NK cells inhibited their cytotoxic function and cytokine secretion. The addition of blocking anti-LLT1 or anti-CD161 mAbs restored NK cell functions, and this independently of MHC class I/KIR or HLA-E/CD94/NKG2A-mediated inhibition (29). LLT1 expression on GC-derived B cell lymphomas therefore dampens NK cell functions and constitutes an immune escape mechanism for these NHLs (**Figure 2**). Consistent with this role, *CLEC2D* was also found among 7 genes associated with resistance of lymphoma and leukemia cell lines to Vγ9Vδ2 T cell-mediated cytotoxicity *in vitro* (83). CD161^+^ αβ and γδ T cells were identified in the TME of FL and GC-DLBCL, along with CD161^-/low^ Tfh cells but their function was not determined (29).

LLT1 was also reported to be expressed at the cell surface of glioma cell lines and primary glioblastoma cells established from freshly resected tumors (84). IHC staining confirmed expression of LLT1 in tumor sections with the level of expression increasing with malignancy grades. LLT1 was also downregulated by treatment with TGF-β *in vitro*. The non-specific mAb clone 4C7 was used but likely detected here LLT1/CLEC2D and not CLEC2A/KACL, as the latter has not been reported to be expressed in these cancers. In addition, downregulation of LLT1 using siRNA targeting *CLEC2D* enhanced NK cell lysis of glioma cells. More recently, Mathewson et al. confirmed by RNA *in situ* hybridization that *CLEC2D* mRNA was detected in the two major classes of diffuse gliomas: isocitrate dehydrogenase mutant glioma (IDH-G) and IDH-wild type glioblastoma (GBM) (65). They used a CRISPR-CAS9 approach to abrogate CD161 in CD8^+^ T cell clones and identified an inhibitory role for CD161 (**Figure 2**).

Besides NHLs and gliomas, LLT1 was reported on hormone-refractory and sensitive prostate cancer cell lines and prostate cancer tissues (85), on triple-negative breast cancer cell lines (86) and on a colon cancer cell line (87). Expression of LLT1 by these cancer cell lines inhibited NK cell-mediated cytotoxicity which was restored by addition of blocking anti-LLT1 mAb or inactivation of *CLEC2D* gene (**Figure 2**).

LLT1 was also reported expressed in cutaneous squamous cell carcinomas of the head and neck (cSCCHN) (88). However, this study only relied on IHC staining of paraffin-embedded skin tissue sections using the non-specific mAb clone 4C7 which recognizes CLEC2D/LLT1 and CLEC2A/KACL (15, 20). Skin express high levels of CLEC2A/KACL, in particular on keratinocytes (89). As cSCCs originate from transformed keratinocytes, results could be distorted by the detection of CLEC2A. Additional stainings of cSCCs with specific anti-LLT1 mAbs have to be performed to draw conclusions.

Head and neck squamous cell carcinomas are a group of tumors that arise from diverse locations such as oral cavity, oropharynx, hypopharynx and larynx. LLT1 expression was detected in oropharyngeal SCC (OPSCC) which were negative for the human papilloma virus (HPV) (90). Again, the clone 4C7 was used in this study. As CLEC2A/KACL expression has not been reported outside of the skin, 4C7 may only detect LLT1 in these patients but results need to be confirmed.

### LLT1 is Detected on Infiltrating Immune Cells and is a Marker of Tertiary Lymphoid Structures

Besides expression on tumor cells, *CLEC2D* mRNA and LLT1 protein have been detected in immune cells within the TME. This is not surprising considering that LLT1 is expressed on activated hematopoietic cells. *CLEC2D* mRNA was found in myeloid cells from GBM and IDH-G diffuse gliomas scRNA-seq datasets (65). LLT1 protein was detected on B and T cells infiltrating HPV^+^ OPSCC (62) and on B and T lymphocytes within the stroma of non-small cell lung cancer (NSCLC) but not in adjacent lung tissue (54). In HPV^-^ OPSCC, LLT1 was detected on tumors and on TILs (90). Based on the IHC stainings, the study showed that the HPV^-^ OPSCC patients with LLT1^+^ tumors and low density of LLT1^+^ TILs had the worst prognostic while patients with LLT1^-^ tumors and high density of LLT1^+^ TILs had the highest survival rate. This is consistent with the demonstration that strong lymphocytic infiltration of solid tumors is associated with good clinical outcome (91).

Importantly, LLT1 was found primarily associated with ectopic lymphoid organizations called Tertiary Lymphoid Structures (TLS) in NSCLC patients (54) (**Figure 2**). The development of TLS is associated with chronic inflammation, infection, and their presence in the TME has been associated with better clinical outcome in many cancers (92–94). TLS exhibit the same organization as SLO, with a T cell zone characterized by T cells forming clusters with mature DC, adjacent to a B cell zone characterized by an active GC containing FDC and Tfh. Staining of NSCLC sections revealed that high LLT1 expression colocalized with CD20, at the vicinity of CD21^+^ FDC within GC. A moderate but positive correlation between LLT1^+^ and CD21^+^ follicles indicate that LLT1 is a marker of active TLS. These mature TLS have been shown to function as sites where local antitumor adaptive immune responses develop (92, 94, 95). The role of LLT1 in these responses is still to be fully explored but it may have similar function as LLT1 expressed by GC B in SLOs, and one can postulate that LLT1/CD161 interaction may participate to the T-B cell interaction leading to the development of specific and protective antitumor B-cell and T-cell immune responses. Of note, CD161 is highly expressed on ILCs among which the ILC3 subset was suggested to play a crucial role in TLS formation (96, 97). This observation suggests that LLT1/CD161 may also participate to the formation of TLS.

### Expression of CD161 is Associated with Good Prognosis in Most Cancers

The meta-analysis by Gentles et al. (79) put forward a role for CD161 in cancer. In this study, *KLRB1* was found to be the gene most frequently associated with favorable clinical outcome in ~18 000 human tumors across 39 malignancies. Further analysis of *KLRB1* RNA expression and clinical data from The Cancer Genome Atlas (TCGA) and the Genotype-Tissue Expression (GTEx) indicate that it is upregulated in most human cancers but also downregulated in others (98).

CD161 is expressed by NK cells which contribute to antitumor responses by direct cytotoxicity and cytokine production, promoting adaptive immune responses. NK cells have been implicated not only in the control of primary tumors (99), more prominently at early stages (100), but also in the control of metastasis (101–103). The detection of LLT1 on tumor cells and the inhibitory signal delivered to NK cells by CD161 engagement can lead these tumors to escape from NK cell control (**Figure 2**). To what extend LLT1/CD161 interaction contributes to tumor and metastatic subversion of NK cell surveillance needs to be assessed.

CD161 is also expressed by T cells. A few studies have phenotyped CD161^+^ T cells infiltrating solid tumors and CD161 was found expressed primarily on CD4^+^ T cells, CD8^+^ T cells and γδ T cells (**Figure 2**). The role of LLT1/CD161 interaction within the TME is still not fully understood and seems to vary among cancers. An early study investigated the phenotype and function of T cells expressing CD161 in the blood, within TILs and in malignant effusions from patients with breast, ovarian, lung, colon, pancreas and stomach cancers (104). They reported an increased frequency of polyclonal CD161^+^CD4^+^ T cells that retained CD28 expression and secreted the Th1 cytokine IFN-γ but also Th2 and suppressive cytokines such as IL-4, IL-10 and TGF-β. In NSCLC patients, the phenotyping of CD161^+^ T cells identified a higher frequency of CD161-expressing CD4^+^ T cells infiltrating the tumor compared to distant lung, draining lymph node and blood (54). These CD161^+^CD4^+^ T cells were mostly conventional T cells and low frequencies of regulatory T cells and Tfh expressed CD161. A moderate increase of the frequency of CD161-expressing CD8^+^ T cells was also observed within the tumor as compared to blood, and this increase concerned mainly CD161^+/dim^ conventional CD8^+^ T cells, CD161^bright^ CD8^+^ MAITs cells being poorly represented. Gene expression profiling of CD161^+^CD4^+^ T cells isolated from NSCLC tumors identified a link between CD161 and T cell activation, co-stimulation and differentiation. Further phenotyping and functional studies revealed that CD161^+^CD4^+^ T cells displayed an effector-memory phenotype and secreted IFN-γ and TNF-α. Compared to CD161^-^CD4^+^ T cells, CD161^+^CD4^+^ T cells from NSCLC tumors expressed higher frequencies of CD69, CD96, CD30L, OX40, PD-1-positive cells and lower frequencies of 4-1BB, CD27 and Tim-3. Such observation suggests that CD161 expression is associated with an activated but not terminally exhausted phenotype. The coexpression of CD161 with OX40 and lack of expression of 4-1BB also suggests that they can both play a critical role in the establishment of a robust CD4 memory pool of tumor-specific T cells that can vigorously respond upon rechallenge (105). Besides, the functional analysis of CD161^+^CD8^+^ T cells compared to CD161^-^CD8^+^ T cells showed that CD161^+^CD8^+^ T cells were less cytotoxic and displayed an exhausted phenotype with higher frequencies of PD-1 and Tim-3-positive cells. Nearly all of them also expressed the tissue resident CD103 integrin which is thought to promote retention of T cells in epithelial tumor islets (106). Altogether, the analysis of CD161^+^CD4^+^ and CD161^+^CD8^+^ T cells within NSLCL tumors, and the detection of LLT1 expression primarily within TLS, strongly suggests that LLT1/CD161 interaction plays a critical role in immune surveillance of NSCLC. Corroborating this, processing of public databases identified *CLEC2D* and *KLRB1* association with favorable clinical outcome in NSCLC (54).

The study of TILs from HPV^+^ and HPV^-^ OPSCC also identified high frequencies of CD161^+^ effector memory CD4^+^ and CD8^+^ T cells which were associated with better overall survival (107). Recently, further in-depth analysis showed that only effector-memory CD161^+^CD4^+^ T cells were associated with better survival of HPV^+^ OPSCC patients (62). The expansion and stimulation of HPV-specific CD161^+^CD4^+^ T cell clones revealed a Th1 phenotype with secretion of IFN-γ and TNF-α. Expression of CD161 was associated with a stronger response to suboptimal antigen stimulation. Nevertheless, in this study, *in vitro* co-engagement of CD161 with CD3 and/or CD28 did not lead to any consistent modulation of the secretion of IFN-γ. Similarly, no significant modulation of IFN-γ and TNF-α secretion was detected when CD161^+^CD4^+^ T cell clones were stimulated with autologous B cells expressing LLT1 in the presence of blocking anti-CD161 mAb or when CD161 expression was abrogated in T cells using a CRISPR-Cas9 approach. Importantly, LLT1 was shown to be upregulated on the activated CD161^+^CD4^+^ T cell clones and this expression in cis was gradually lost over time. Such modulation may disrupt assays and complicate the analysis of the signals delivered by CD161. In addition, CD161 expression was also downregulated by TCR triggering and its downregulation was potentiated by addition of TGF-β. Studies of the expression of transcription factors identified the transcriptional transactivator SOX4 specifically expressed in CD161^+^ CD4^+^ T cells infiltrating HPV^+^ OPSCC. Such findings suggest that CD161 is involved in amplifying TCR signal, as opposed to PD-1 or CD39 which were increased upon TCR ligation and by TGF-β. Altogether, these data are consistent with CD161 playing a role in the activation of CD4^+^ T cells rather than in their inhibition (62).

In other types of cancers, CD161 expression is associated with worst clinical outcome. This has been shown for early-relapse hepatocellular carcinomas (108) and glioblastomas (65, 109). Mathewson et al. (65) deciphered the role of CD161 on TILs in gliomas. They performed RNAseq analysis of TILs from resected gliomas and identified highly cytotoxic CD8^+^ T cells expressing *KLRB1*, as well as conventional CD4^+^ T cells. These cells expressed low level of PD-1. Flow cytometry staining also showed that a large fraction of glioma-infiltrated CD8^+^ and CD4^+^ T cells expressed CD161 while only small proportions were present in the blood of the same patients. Interestingly, their frequencies are much higher than those detected in NSCLC and OPSCC highlighting that there is heterogeneity in frequencies and phenotypes among cancer types. Then, they examined the function of CD161 which could interact with LLT1 detected on glioma tumor cells (65, 84). They set up *in vitro* co-cultures of HLA-A*02.01^+^ patient-derived gliomas and cell lines expressing the tumor antigen peptide NY-ESO-1, with engineered T cells that express a NY-ESO-1 specific HLA-A*02.01-restricted TCR, and either expressed or lacked CD161, deleted using a CRISPR-Cas9 approach. These co-cultures showed that the abrogation of CD161 expression enhanced T cell-mediated cytotoxicity and cytokine secretion, revealing an inhibitory role for CD161 in these CD8^+^ T cells. Such function was also confirmed *in vivo* using two humanized mouse models. Inactivation of *KLRB1* gene in T cells transferred in the brain of glioma-bearing mice slowed down tumor growth. These results are also consistent with a recent study analyzing RNAseq data from 916 human glioblastoma samples which showed that CD161 is a biomarker of a particularly aggressive subtype, the mesenchymal gliomas. And CD161 is significantly associated with the higher grades of glioblastomas (109).

Several explanations could be put forward to explain the discrepancies between studies. First, the role of CD161 may differ between cancer types. While CD161 is associated with favorable clinical outcome in most cancers, in others, it is associated with a poor outcome. CD161^+^ CD8^+^ T cells infiltrating early-relapse hepatocellular carcinomas are reported to be significantly associated with a higher second recurrence rate (108) and glioma patients with higher expression of CD161 have significantly shorter overall survival (109). Differences between cancers may originate from variable frequencies of CD161 expressing CD4^+^ and CD8^+^ TILs, as observed between glioblastomas, NSCLC and OPSCC or they may originate from heterogeneity of the TME and antitumor immune responses. Second, the role of CD161 may differ between subsets of CD4^+^ and CD8^+^ T cells. If CD161, like other NKRs regulate T cell activation, the signals delivered may depend on the overall threshold of activation which differ among subsets of CD4^+^ and CD8^+^ T cells. It is interesting to note that in some studies, CD161 expressing CD8^+^ T cells displayed an innate-like memory phenotype with low cytotoxicity and immunosuppressive functions, consistent with CD161 delivering inhibitory signals (49, 54, 108). Gene expression comparisons have demonstrated that CD8^+^ T cells are more closely related to NK cells than to CD4^+^ T cells (49, 110). In this context, one could speculate that CD161 preferentially deliver inhibitory signals in NK and innate-like CD8^+^ T cells. Differences between subsets of CD4^+^ and CD8^+^ T cells may also depend on their spatial localization and their interaction with LLT1-expressing tumor and/or immune cells. Indeed, CD8^+^ TILS are more frequently found within tumor islets as evidenced by CD103 expression compared to CD4^+^ T cells (106). Third, the upregulation of LLT1 upon activation of T cells may modulate TCR activation threshold. This LLT1/CD161 *cis* interaction would only occur in the control-edited T cells and not in the KLRB1-edited T cells (65) and would be variably detected in T cell clones and lines used in *in vitro* stimulations (27, 45, 54, 62, 63, 111). Fourth, the CRISPR-Cas9 approach involves steps of transfection and selection of clones. Selected control-edited T cells therefore differ from KLRB1-edited T cells and TCR activation threshold may not be comparable. Fifth, metabolic programming control T cell functions and may be influenced not only by the nature of T cells, the strength of the signal delivered but also the medium used in the *in vitro* assays (112).

In conclusion, further work has to be performed to understand the role played by CD161 on CD8^+^ and CD4^+^ T cells infiltrating human tumors. Studies so far point towards a preferential expression of CD161 on tumor specific T cells and its interaction with LLT1 expressed by tumors cells and/or B cells within TLS.

## LLT1 and CD161 are Emerging as Attractive Biomarkers and Targets in Cancer Immunotherapy

Tumor development is associated with immune suppression. To improve success rates of current immunotherapies, one needs to resolve immunoresistance mechanisms. Constant improvement in the understanding of antitumor immune responses brings clues to overcome such issues. The studies on LLT1 and CD161 provide a rational for their use both as biomarkers of ongoing antitumor B cell and T cell responses and as potential targets in cancer immunotherapy.

### LLT1 and CD161 as Biomarkers of Ongoing Antitumor B Cell and T Cell Responses

Considering LLT1 and CD161 as biomarkers in cancer is particularly relevant since T cells are major actors of the antitumor immune response and B cells are emerging as important players as well. The presence of LLT1 on GC B cells in active TLS (54) suggests that LLT1 contributes to antitumor immune responses. Indeed, the presence and density of TLS has been correlated with favorable prognosis in many cancers (93, 113, 114). The maturity of TLS is of importance, GC-containing TLS being the most mature and best predictors of lack of cancer recurrence (115). In addition, the presence of TLS within the TME has recently been associated with therapeutic responses to immune checkpoint immunotherapies and to lower recurrence (116–121). The analysis of gene expression and immune cell composition of the TME in more than 600 samples of soft-tissue sarcoma identified a sub-group characterized by the presence of TLS that contained T cells and FDC and were particularly rich in B cells. This sub-group with the strongest B-cell signature was also associated with the best response rate to anti-PD-1 treatment (116). Similarly, B cell and TLS signatures as well as T effector signatures predicted responses to anti-PD-1 and anti-CTLA-4 antibodies in melanomas and renal cell carcinomas (117, 120). This suggests that B cells play a significant role in shaping protective T cell responses. The detection of LLT1 on GC B cells in these active mature TLS therefore supports a prognostic role of LLT1, consistent with the reported association with favorable outcome in NSCLC (54). TLS^high^ tumors have been characterized by an increased proportion of activated effector/memory CD8^+^ T cells and early differentiated, activated and non-regulatory CD4^+^ T cells (122, 123). CD161 being expressed on effector/memory CD4^+^ and CD8^+^ T cells and being associated with better clinical outcome (54, 79), these observations highlight the relevance of LLT1 and CD161 as biomarkers of active antitumor B cell and T cell immune responses.

### Targeting LLT1 and CD161 to Improve Antitumor Responses

The goal of immunotherapies in cancer treatment is to trigger or restore efficient antitumor immune responses. LLT1 and CD161 regulate such responses, so, strategies targeting their interaction could be designed to stimulate immune surveillance. In cancers where LLT1 is expressed by the tumor cells, LLT1 may be targeted as tumor-associated antigen. It could therefore be relevant to develop therapeutic mAbs against LLT1 that could trigger antibody-dependent cellular cytotoxicity (ADCC), antibody-dependent cellular phagocytosis (ADCP), complement-dependent cytotoxicity (CDC) or bispecific antibodies and CAR-NK or CAR-T cells to promote tumor cell elimination. When LLT1 is expressed by tumor cells, blocking LLT1/CD161 interaction could also prevent the inhibition of NK cell functions and unleash their antitumor activity. This would restore antitumor cytotoxic activity of NK cells in primary tumors but also strengthen their control of metastatic disease (102). Such therapeutic approach has provided promising results with the use of immune checkpoint inhibitors targeting inhibitory NKRs like NKG2A (124, 125) or co-inhibitory receptors like TIGIT, LAG-3 and TIM-3 (126, 127). Regarding T cells, the challenge will be to assess whether the interaction could positively or negatively impact cancer progression, as the function of CD161 as a co-stimulatory or co-inhibitory receptor on effector and memory cells is still a matter of debate. In the first case, blocking the interaction may deprive T cells of an additional beneficial second signal in support for example of a weak TCR signaling. In the second case, it may further increase cytotoxic functions and cytokine production of T cells. Recent progress of spatial high dimensional imaging technologies, genomic and proteomic technologies and 3D tumor models will certainly help to tackle these issues (128–130). Alternatively, targeting LLT1/CD161 interaction in the TME may improve strategies aimed at stimulating more robust antitumor T cell responses such as immune checkpoint inhibitors, therapeutic cancer vaccines and CAR-T cells or CAR-NK cells. Finally, given the positive association of TLS with improved clinical outcome and better response to immune checkpoint inhibitors, strategies that stimulate the induction of TLS formation could improve antitumor immune responses. Whether targeting LLT1 and CD161 modulate TLS neogenesis, maturation and function will need to be investigated.

## Concluding Remarks

Recent progress on the biology of LLT1 and CD161, together with cancer signatures obtained from gene and protein expression profiling, reinforce the hypothesis that these two receptors are important players that shape antitumor immune responses and influence tumor development and progression. Many questions subsist and a number of mechanisms remain to be tackled, but the fast advances in the understanding of the complexity of the TME provide hope in front of these challenges.

## Author Contributions

VB, CG, and FA wrote the manuscript, which was amended and validated by all the authors. All authors contributed to the article and approved the submitted version.

## Funding

This research was supported by Centre National de la Recherche Scientifique; Institut national de la santé et de la recherche médicale; Université Côte d’Azur; Cancéropole PACA; Région Provence-Alpes-Côte d’Azur; Fondation ARC pour la recherche sur le Cancer; Ligue Nationale contre le Cancer; French Government (National Research Agency, ANR) through the “Investments for the Future” programs LABEX SIGNALIFE ANR-11-LABX-0028 and IDEX UCAJedi ANR-15-IDEX-01.

## Conflict of Interest

CG is full-time employee of Biomunex Pharmaceuticals.

The remaining authors declare that the research was conducted in the absence of any commercial or financial relationships that could be construed as a potential conflict of interest.

## Publisher’s Note

All claims expressed in this article are solely those of the authors and do not necessarily represent those of their affiliated organizations, or those of the publisher, the editors and the reviewers. Any product that may be evaluated in this article, or claim that may be made by its manufacturer, is not guaranteed or endorsed by the publisher.
